# Antibacterial and Antibiofilm Activities of Bluestem (*Andropogon* spp.) Extracts against *Vibrio cholerae*

**DOI:** 10.1155/ijm/9982329

**Published:** 2026-05-03

**Authors:** Jant Cres Caigoy, Tadashi Shimamoto, Tran Dang Xuan, Nguyen Van Quan

**Affiliations:** ^1^ Program of Food and AgriLife Science, Graduate School of Integrated Sciences for Life, Hiroshima University, Higashihiroshima, Japan, hiroshima-u.ac.jp; ^2^ Program of Bioresource Science, Graduate School of Integrated Sciences for Life, Hiroshima University, Higashihiroshima, Japan, hiroshima-u.ac.jp; ^3^ Center for the Planetary Health and Innovation Science (PHIS), The IDEC Institute, Hiroshima University, Higashihiroshima, Japan, hiroshima-u.ac.jp; ^4^ Smart Agriculture Faculty, Graduate School of Innovation and Practice for Smart Society, Hiroshima University, Higashihiroshima, Japan, hiroshima-u.ac.jp; ^5^ Department of Pharmacy, Thai Nguyen University of Medicine and Pharmacy, Thai Nguyen, Vietnam, tnu.edu.vn

**Keywords:** *Andropogon* spp., biofilm inhibition, bluestem, flavonoid glycoside, quorum quenching, *Vibrio cholerae*

## Abstract

*Vibrio cholerae* is a gram‐negative bacterium found in water, particularly in brackish and estuarine environments, and causes cholera—an acute disease transmitted through contaminated food or water. Biofilm formation in *V. cholerae* enhances its resistance to antimicrobial agents and protection against host immune responses. Natural plant extracts have gained attention for their potential antibiofilm properties against human pathogens such as *V. cholerae*. This study evaluates the antibacterial and antibiofilm activities of bluestem (*Andropogon* spp.) extracts against pathogenic *V. cholerae* strains using antibacterial assays, biofilm inhibition and eradication assays, and gene expression analysis. Two extracts, denoted as EAV1 and EAV2, primarily composed of two flavonoid C‐glycosides species, demonstrated significant bactericidal effects on *V. cholerae*. Sub‐MIC levels of the extracts also inhibited the growth of biofilms. Gene expression analysis revealed downregulation of genes encoding biofilm transcriptional regulators, *Vibrio* polysaccharide, and biofilm matrix proteins upon EAV treatment. Results indicate a potential quorum‐quenching activity by targeting the transcription of biofilm regulators and biofilm matrix genes. These findings suggest that *Andropogon* spp. extract is a promising antibacterial and antibiofilm agent against *V. cholerae*, highlighting the potential of plant species with invasive tendencies as sources of antibacterial and antibiofilm compounds against clinically important pathogens.

## 1. Introduction

*Vibrio cholerae*, the causative agent of cholera, is a gram‐negative bacterium that naturally inhabits aquatic environments, particularly freshwater areas. Among the 200 serogroups identified under this species, only the O1 and O139 serogroups are considered as pathogenic due to their ability to produce cholera toxin. The ongoing 7th cholera pandemic (1961 to present) is associated with *V. cholerae* O1 El Tor strains [1]. Some non‐O1/non‐O139 environmental strains have also been reported to cause acute watery diarrhea and cholera‐like infections in humans [2]. Cholera infection begins when *V. cholerae* enters the human small intestine. The pathogen then penetrates the mucus lining and colonizes the surface of the epithelial cells, where it forms biofilms [3]. *V. cholerae* is also being shed back to the environment as biofilms through rice‐watery stools from infected patients [4].

*V. cholerae* biofilm formation is primarily regulated by the quorum‐sensing (QS) system [5]. Activation of the QS system at low cell density (LCD) triggers the biofilm transcriptional regulators AphA, VpsT, and VpsR. These regulators are responsible for the production of extracellular polysaccharides (EPS), known as *Vibrio* polysaccharides (VPS), and extracellular matrix proteins, which are essential for the initiation and maturation of *V. cholerae* biofilms [6]. Biofilm formation is an important virulence factor for *V. cholerae* as it provides protection against host immune responses and increases resistance to antimicrobial agents [7]. Additionally, biofilm formation enables *V. cholerae* cells to colonize the chitinous carapaces of crustaceans such as copepods, thereby facilitating persistence and survival in aquatic environments [8]. For a clinical perspective, inhibiting biofilm formation is an effective strategy to maintain the susceptibility of pathogens to antimicrobials and simultaneously reduce the virulence and pathogenicity of *V. cholerae*.

Although antibiotic administration can be used for severe cholera cases, overuse of antibiotics in self‐limited cholera infections has led to antibiotic resistance. *V. cholerae* has been documented to show resistance to all classes of antibiotics, and currently, no new antibiotics are being development for *V. cholerae* [9]. One promising strategy is the use of natural compounds derived from plants to combat biofilm formation and virulence production of clinically important bacterial pathogens [10–12]. The antibacterial and antibiofilm activities of these plant extracts have been linked to the secondary metabolites such as alkaloids, flavonoids, phenols, glycosides, steroids, saponins, and terpenoids [13, 14]. The mechanisms of the antibiofilm activity of these plant extracts include (1) antiadhesion to the substrate, (2) inhibition of EPS production, (3) biofilm disruption, and (4) quorum‐quenching (QQ) activity [15].

While most of these bioactive compounds have been reported from medicinal plant species, invasive plant species with no known economic value have also been found to contain bioactive compounds. Invasive alien species (IAS) pose a negative ecological impact on biodiversity and economic losses in agriculture [16, 17]. Despite these drawbacks, IAS are potential sources of biologically active compounds due to the accumulation of certain molecules to help the plant adapt in an altered environment [18]. Extracts from IAS were reported to contain significant amounts of phytonutrients and specialized metabolites such as vitamin C, phenols, flavonoids, nonflavonoids, chlorophylls, and carotenoids [19].

Grasses belonging to the Poaceae family are among the world′s most economically important plant groups, as 33%–40% of Earth′s land area is covered by grasslands, their seeds serve as staple cereals, and their vegetative parts are used as fodder for cattle and other herbivores [20]. The bluestems and broomsedge (*Andropogon* spp.) are clump‐forming, warm‐season grasses in the Poaceae family and are often used for erosion control and meadow plantings [21]. This genus is distinctly characterized by its hair‐like stems and male‐only spikelets. Although native to eastern North America, bluestems, particularly the broomsedge bluestem (*A. virginicus* L.), have been introduced and proliferated in Hawaii, Central and South America, New Zealand, Australia, and Japan [22]. In Japan*, A. virginicus* is identified as an invasive species and is distributed from the Kyushu region to the Kanto region [23]. It typically grows 0.5–1.5 m tall, with erect culms, linear leaves (10–40 cm long, 2.6 mm wide), and a broom‐like inflorescence of paired racemes bearing long awned spikelets [24]. *A. virginicus* invades plant communities in nutrient‐deficient soil and commonly dominates roadsides, old fields, and pastures [25].

Biological activities of some plant species under the Poaceae family have already been reported. For example, extracts from *Cynodon dactylon* (bermuda grass) showed strong antibacterial activity against *Staphylococcus aureus* [26]. Lemon grass (*Cymbopogon citratus*; formerly known as *Andropogon citratus*) essential oils have shown antibiofilm activity against multidrug‐resistant *V. parahaemolyticus* [27]. Root decoction of *A. virginicus* is traditionally used to relieve backaches, whereas leaf decoctions are taken as a remedy for diarrhea or used to wash frostbite, sores, and itching [28]. Diverse biological activities such as antioxidant, antityrosinase, antiamylase, and cytotoxic activities from *A. virginicus* extracts have been reported [29].

Plant extracts exhibiting strong antioxidant activities are also associated with antibacterial properties [30]. For instance, antioxidant‐rich plant extracts have been reported to interfere with microbial cell membranes [31], inhibit QS, and impair biofilm formation [32]. Therefore, the previously reported antioxidant activity of bluestem species may be indicative of their potential antibacterial and antibiofilm effects. In this study, we demonstrate the antibacterial and antibiofilm activity of *Andropogon* spp. extracts against the pathogenic *V. cholerae.* The results from this study could provide a basis for new drug development against *V. cholerae* and other human pathogens. Furthermore, this study highlights the potential of plant species with invasive tendencies as sources of antibacterial and antibiofilm compounds against clinically important human pathogens.

## 2. Materials and Methods

### 2.1. Plant Materials

Two samples of *Andropogon* spp. were collected at the Higashi‐Hiroshima Campus, Hiroshima University, Hiroshima, Japan, in November 2023. Species identification was based on morphological assessment combined with previously documented descriptions [29]. Based on significant morphological variations and differences in local distribution, two distinct phenotypes of *Andropogon* spp. were selected and designated as EAV1 and EAV2. While EAV1 is predominantly found in sloped terrains, characterized by thinner stems and sparse, isolated growth, EAV2 is distributed in flatter areas, displays thicker stems, and typically grows in dense clumps. Voucher specimens identified as AV1‐N2023QJ and AV2‐N2023QJ were deposited at the Laboratory of Plant Physiology and Biochemistry, IDEC Institute, Hiroshima University, Japan.

### 2.2. Sample Extraction

Aerial parts of AV1 and AV2 were finely chopped, thoroughly rinsed with tap water, soaked in 0.5% NaOCl solution for 4 h, and subsequently washed with distilled water. After draining, samples were dried in a convection oven at 40°C for 6 days. Dried samples were then powdered and stored in sealed zip‐lock bags at 4°C. A 5 g portion of each sample (AV1 or AV2) was extracted with 80 mL of an extraction solvent composed of 80% methanol (Junsei Chemical Co. Ltd., Japan) and 0.5% citric acid (Kanto Chemical Co. Inc., Japan). The mixture was magnetically stirred at room temperature for 4 h and filtered to obtain the first extract. The residue was re‐extracted twice with 80 mL of the same solvent under identical conditions. All filtrates were combined and concentrated under vacuum at 50°C to a final volume of approximately 40 mL, the crude extract in an aqueous phase. This aqueous extract was further subjected to liquid–liquid extraction sequentially with hexane and ethyl acetate (EtOAc), each extraction performed three times at a 1:1 solvent‐to‐extract ratio. The hexane (HAV1, HAV2) and EtOAc (EAV1, EAV2) extracts were evaporated under vacuum to obtain dried extracts. For the subsequent in vitro experiments, the dried extracts were dissolved in absolute ethanol to achieve 10,000 *μ*g/mL. Based on preliminary bioactivity screening (data not shown), only EAV1 and EAV2 exhibited significant activity and were selected for further analysis.

### 2.3. Determination of Total Phenolic and Total Flavonoid Contents (TFCs)

Total phenolic content (TPC) and TFC of EAV1 and EAV2 were determined by the Folin‐Ciocalteu method [33] and aluminum chloride colorimetric method [29]. Briefly, 20 *μ*L of sample was mixed with 100 *μ*L of 10% Folin‐Ciocalteu reagent and 80 *μ*L of 7.5% Na_2_CO_3_. The mixture was incubated at room temperature in the dark for 30 min, and absorbance was measured at 765 nm using a spectrophotometer. Results were expressed as milligrams of gallic acid equivalent per gram of dry weight (mg GAE/g DW). For TFC determination, equal volumes of sample and 2% AlCl_3_ solution were mixed and incubated for 15 min at room temperature in darkness. Absorbance was measured at 430 nm, and results were presented as milligrams of rutin equivalent per gram dry weight (mg RE/g DW).

### 2.4. Identification of Major Compounds

Dominant phytochemical constituents in EAV1 and EAV2 were tentatively identified using LC‐ESI‐MS/MS and HPLC‐UV/VIS analyses. For the initial comparison of major compound contents, an HPLC system (Jasco, Tokyo, Japan), including a PU‐4180 RHPLC pump, LC‐Net II/ADC controller, and UV‐4075 UV/VIS detector, was employed. Separation was conducted on an XBridge BEH Shield RP18 column (130 Å, 5 *μ*m, 2.1 mm × 100 mm, Waters Cooperation, Milford, Massachusetts, United States). The mobile phase consisted of solution A (0.1% aqueous formic acid) and solution B (100% acetonitrile) with the following gradient: 5% B (0–2 min), linear increase from 5% to 70% B (2–12 min), 100% B maintained (12–22 min), returning to 5% B (22–24 min), followed by equilibration for 10 min. The injection volume was 5 *μ*L, with a flow rate of 400 *μ*L/min at room temperature. Samples (0.5 mg/mL) were analyzed with UV detection at 350 nm. Peak areas were integrated using ChromNAV Ver.2 software (JASCO). The dominant peaks appeared at 9.05 and 9.17 min.

LC‐MS/MS procedures followed those previously described by [29]. Briefly, the liquid chromatography conditions, including the stationary phase, gradient mobile phase, sample concentration, and injection volume, were maintained as previously described. The major peaks were identified at 7.35 and 7.57 min. Electrospray ionization (ESI) was operated in negative mode with the following parameters: ion source voltage at 4.5 kV, capillary voltage at −50 V, tube lens offset at −80 V, and capillary temperature at 350°C. Nitrogen was used as the carrier gas, with sheath and auxiliary gas flow rates set at 60 and 20 arbitrary units, respectively. Mass spectra were recorded at a resolution of 60,000 across a scan range of *m*/*z* 115–2000. Full‐scan and data‐dependent MS/MS spectra of deprotonated molecular ions were acquired and processed using Xcalibur software integrated with the NIST 20 database. Additional spectral information was referenced using online databases such as MassBank and PubChem.

### 2.5. Bacterial Strains and Growth Conditions

*V. cholerae* strains were selected according to (1) their pathogenic serogroup, (2) HapR variation, and (3) ability to produce biofilms (Table 1). HapR variations in *V. cholerae* significantly affects biofilm formation where HapR negative strains produce more biofilm due to a nonfunctional biofilm repressor under high‐cell density [34]. To account for this genetic variation, we included both HapR‐positive and HapR‐negative strains in this study. *V. cholerae* strains were routinely cultured from −80°C glycerol stock to LB agar medium at 37°C for 24 h. Single colonies were picked and inoculated into test tubes containing 5 mL LB broth. Cells were then aerobically cultured in a shaking water bath (150 rpm) at 37°C for 24 h. All experiments were performed in a BSL2 laboratory.

**Table 1:** *V. cholerae* strains used in this study.

Strain	Serogroup	Biotype	HapR variation	Remarks
P1418	O1	El Tor	Frameshift, HapR^neg^	*ctx* ^+^
14‐9/9	O1	El Tor	Intact, HapR^pos^	*ctx* ^+^
MO20	O139	—	Intact, HapR^pos^	*ctx* ^+^
B0311	O139	—	Frameshift, HapR^neg^	*ctx* ^+^, rugose colony

### 2.6. Determination of Antibacterial Activity of *A. virginicus* Extracts

The MIC of the EAV1 and EAV2 extracts was determined using the broth microdilution method [35]. Overnight *V. cholerae* cultures were centrifuged (150,000 rpm, 1 min) and resuspended in saline solution (0.85% NaCl) to achieve a 0.5 McFarland standard (8 log CFU/mL). In this study, the biofilm formation assay for *V. cholerae* was optimized under M9 conditions. Hence, we used the M9 minimal medium for the MIC/MBC analysis. A two‐fold serial dilution of M9 broth supplemented with AV extract from 250 *μ*g/mL was performed in a 96‐well microtiter plate. In a 100 *μ*L M9 minimal medium, 5 *μ*L of the 0.5 McFarland standard was inoculated. The plates were then incubated at 37°C for 16–18 h. After incubation, 10 *μ*L from each well was spotted on LB agar plates. The agar plates were then incubated at 37°C for 24 h. The MBC was determined as the lowest concentration of the AV extracts that exhibited no colony growth on the LB agar plates [36]. The MIC was determined as the lowest concentration to inhibit the growth of *V. cholerae* compared with the negative control (0 *μ*g/mL) [36]. A control treatment with ethanol (final concentration at 2.5%) corresponding to the highest extract concentration (250 *μ*g/mL) was also used. MIC and MBC analyses were performed three times in duplicates.

### 2.7. Determination of Minimum Biofilm Inhibitory Concentration (MBIC)

Overnight cultures of *V. cholerae* grown in LB broth were centrifuges (150,000 rpm, 1 min) and resuspended in 0.85% saline solution to achieve an OD_600_:0.5. The inoculum was added into the M9 minimal medium (1:100 dilution) containing 250, 125, 62.5, 31.25, 15.6, 7.8, 3.9, and 1.95 *μ*g/mL extracts. Aliquot of 200 *μ*L was transferred to a V‐well 96‐well microtiter plate and the plates were incubated at 37°C for 24 h under static aerobic conditions.

Attached biofilms grown in the microtiter plates were quantified based on the crystal violet staining method by O′Toole [37]. Briefly, planktonic cells were removed from the wells and the wells were washed three times with 200 *μ*L distilled water. Thereafter, the bound biofilms were stained with 0.1% aqueous crystal violet solution for 10 min. Excess stain was removed and the wells were washed again with distilled water three times. The bound stain was solubilized with 200 *μ*L 30% aqueous acetic acid solution. An aliquot of 150 *μ*L was then transferred to a flat‐bottom microtiter plate and the relative biofilm was measured at 570 nm using a microplate reader (MultiSkan Sky, Thermo Scientific, Sweden). M9 minimal medium with 2.5% ethanol (OD_control_) was used as negative control while M9 minimal medium without inoculation served as the blank control (OD_blank_). The experiment was performed in three independent runs with four biological replicates. The percent (%) biofilm reduction was calculated using the formula:

%Biofilm reduction=100×ODcontrol−ODblank−ODtreatment−ODblankOsDcontrol−ODblank

From the percentage biofilm reduction results, the MBIC_50_ was determined as the AV extract concentration exhibiting at least 50% biofilm reduction compared with the negative control (0 *μ*g/mL; 2.5% ethanol). In addition, the half‐maximal (50%) inhibitory concentration (IC_50_) was also estimated using the formula [38]:

IC50=10logAB×50−CD−C+logB

where *A* is the corresponding concentrations of test compound directly above 50% inhibition, *B* is the corresponding concentrations of test compound directly below 50% inhibition, *C* is the % inhibition directly below 50% inhibition, and *D* is the % inhibition directly above 50% inhibition.

### 2.8. Determination of Minimum Biofilm Eradication Concentration (MBEC)

The MBEC assay was performed according to Tsukatani et al. [39]. Briefly, *V. cholerae* bacterial suspension in 0.85% saline solution (OD_600_:0.5) was diluted (1:100) in M9 minimal medium. An aliquot of 180 *μ*L inoculated M9 medium was added to each well of a 96‐well flat‐bottomed microplate. The plate was covered with a sterile 96‐pin microtiter plate lid (Sansyo, Japan) and incubated at 37°C for 24 h to allow biofilms to form. After incubation, the peg lids with established biofilms were transferred into new 96‐well flat‐bottomed microtiter plates, with each well containing 200 *μ*L M9 medium supplemented with EAV extracts. The plates were incubated again at 37°C for 24 h. The amount of biofilm formed was measured as described above. The MBEC_50_ was determined as the extract concentration exhibiting at least 50% reduction of the 24‐h grown biofilm.

### 2.9. RNA Extraction

*V. cholerae* cells were grown for 24 h in M9 minimal medium with either EAV1 or EAV2 extracts supplemented at their respective 0.75× MIC. Cells were harvested by centrifugation (130,000 rpm, 3 min, 4°C). Total RNA was extracted using the Trizol reagent according to the manufacturer′s instructions. The RNA extracts were then subjected to DNase treatment (DNase I Recombinant, Roche, Sigma‐Aldrich, Germany). RNA concentration and quality were checked by Nanodrop (BioSpec‐nano, Shimadzu, Japan). cDNA was prepared from 2 *μ*g of DNase‐treated RNA using ReverTra Ace cDNA synthesis kit (Toyobo, Japan). cDNA synthesis was performed immediately to avoid RNA degradation.

### 2.10. Gene Expression Analysis

Primers used in gene expression analysis are listed in Table 2. Real‐time PCR was performed using a StepOnePlus Real‐Time PCR system (Applied Biosystems, Singapore). A relative gene expression was performed using SYBR Green PCR kit (SYBR Green qPCR Master Mix, Thermo Scientific, Japan) with cDNA (1:10 dilution) as the template. Reactions (10 *μ*L per sample) were performed in duplicate. All reactions contained 20 ng cDNA, 5 *μ*L of SYBR Green Master mix, 0.4 *μ*L of each primer (10 *μ*M), and sterile water. The *gyrB* gene was used to normalize transcript levels. PCR experiments were performed three times in duplicates.

**Table 2:** Primers used for gene expression analysis.

Target	Primer name	Sequence	Reference
*aphA*	AphA‐F	ATC GCG TAA ATT GGT CGC TCA C	[40]
	AphA‐R	AAT CCA TGC TTG GCG AAC CAG	[40]
*vpsT*	vpsT‐RTF	CGC AGG ATA TTG AGC ATA AGC	[41]
	vpsT‐RTR	CGG CAC GAT AAT GGA GAA TG	[41]
*vpsR*	vpsR‐F	GAG TCT CAG CTC GAT CTT CC	This study
	vpsR‐R	CGA TAC TCC CAG CTC TTT GG	This study
*vpsU*	VpsU‐F	CTT AGC AAG GCG AAT CGA CAA G	[40]
	VpsU‐R	CGC ATT TAT CCC CTG CTC TTG	[40]
*vpsL*	VpsL‐F	AAT CGC TAC ATG CTG CGT CAC	[40]
	VpsL‐R	CAG CGA TGG ATG TAG TCC AGA TC	[40]
*rbmA*	RbmA‐F	TTC CTG TCA ATG CGA GAG AG	[40]
	Rbma‐R	CTC GCT CAC CAG AAA CAA TGT C	[40]
*bap1*	Bap1‐F	CCA CGG TGT GTT TGT GTA TGA G	[40]
	Bap1‐R	CTG TTG TGG GTT AAC CAG CTT GG	[40]
*gyrB*	gyrB‐F1	GGA TTG GCT GAT CAA AGA GTC G	[40]
	gyrB‐R1	TCC ATC GTA GTT TCC CAC AGC	[40]

### 2.11. Motility Assay

Swimming motility assay was performed using M9 minimal medium with 0.3% agar containing 0.75× MIC of the EAV extract. A control motility agar medium, supplemented with an equivalent amount of ethanol, was used. Overnight LB cultures were stabbed using a toothpick into the motility agar medium. Plates were then incubated at 37°C for 12 h, and the swimming motility diameter was measured. The experiment was performed three times with at least three replicates each.

### 2.12. Microscopic Analysis

*V. cholerae* biofilms were grown on glass coverslips in M9 minimal medium overnight at 37°C. Subsequently, preformed biofilms were treated with EAV extracts at 0.75× MIC. For compound light microscopy, biofilms on glass coverslips were stained with 0.1% aqueous crystal violet solution for 10 min. Biofilms were then rinsed with distilled water to remove excess stain and observed under a light microscope at 1,000× magnification (Olympus CH40, Japan).

Biofilm samples were prepared for scanning electron microscopy (SEM) as follows. Samples were fixed in 2.5% glutaraldehyde in 0.1 M phosphate buffer (pH 7.4) at 4°C for 24 h, then washed three times with the same buffer for 10 min each. Postfixation was performed in 2% osmium tetroxide for 30 min at room temperature, followed by three additional 10‐min washes with phosphate buffer. Samples were dehydrated through a graded ethanol series of 30%, 50%, 70%, 90%, and three changes of 100% ethanol, each for 10 min, and air‐dried. The dried samples were mounted and sputter‐coated with platinum using a JFC‐1600 automatic sputter coater (JEOL, Japan). SEM observation was conducted using a JSM‐5610 LV scanning electron microscope (JEOL, Japan) at an accelerating voltage of 10 kV.

### 2.13. Statistical Analysis

Statistical significance was determined using analysis of variance (ANOVA) set at *α* = 0.05. Post hoc analysis was performed using Duncan′s multiple range test. For gene expression results, Student′s *t*‐test was used to compare between the control and EAV‐treated samples. All statistical analyses were performed in R studio software.

## 3. Results and Discussions

### 3.1. Determination of TPC and TFC

As presented in Table 3, the TPC in EAV1 (5.87 *μ*g GAE/g DW) was higher than that in EAV2 (4.55 *μ*g GAE/g DW). Similarly, TFC values were significantly higher in EAV1 (3.36 *μ*g RE/g DW) compared with EAV2 (2.40 *μ*g RE/g DW).

**Table 3:** Total phenolic and flavonoid contents of EAV1 and EAV2.

Sample	TPC (*μ*gGAE/g DW)	TFC (*μ*gRE/g DW)
EAV1	5.87 ± 0.10	3.36 ± 0.49
EAV2	4.55 ± 0.20	2.40 ± 0.36

### 3.2. Comparison of Major Compounds

**Figure 1:**
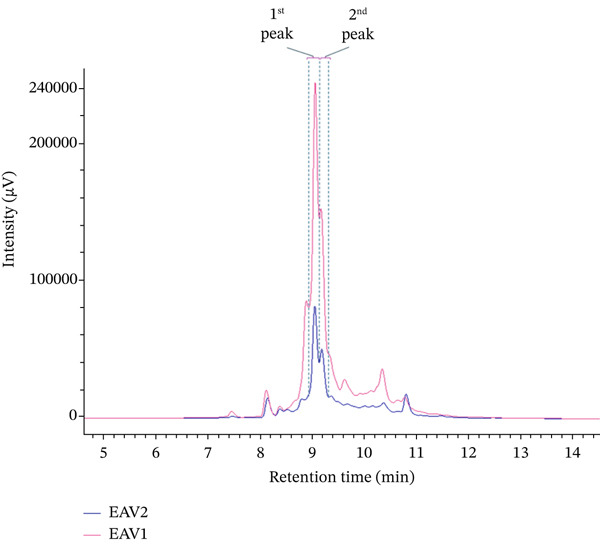
HPLC‐UV chromatogram of EAV1 and EAV2 at a concentration of 0.5 mg/mL. Two peaks corresponding to flavonoid C‐glycosides were detected at 9.05 min (1st peak) and 9.17 min (2nd peak).

Two major flavonoid glycoside compounds were detected at retention times of 7.35 and 7.57 min by HPLC‐ESI‐MS/MS (Figure S1 and S2), which correspond to the major chromatographic peaks at 9.05 and 9.17 min by HPLC‐UV/VIS (Figure 1), respectively. The first compound was identified by a precursor ion [2 M‐H]^−^ at *m*/*z* 863.20, whereas the second compound displayed a precursor ion [M − H]^−^ at *m*/*z* 431.10. Fragmentation patterns suggested that these compounds are likely isomers, sharing a calculated exact mass of 432.1 g/mol and molecular formula C_21_H_20_O_10_. Analysis of dominant ion fragments further supports this identification: The ion at *m*/*z* 327 may result from cross‐ring cleavage of a *C*‐glycosidic sugar moiety (commonly observed in flavone *C*‐glycosides), whereas the ion at *m*/*z* 357 is likely another cross‐ring cleavage fragment, indicating the presence of a *C*‐glucoside moiety [42]. These findings suggest that the detected compounds are likely flavonoid *C*‐glycosides. The observation of a stable dimeric ion at *m*/*z* 863.20 ([2 M–H]^−^) is consistent with previous high‐resolution UHPLC‐MS studies. For example, An et al. (2025) [43] reported aloe‐emodin‐8‐O‐*β*‐D‐glucopyranoside (C21H20O10) displaying a precursor ion at *m*/*z* 431.10 ([M–H]^−^) along with a corresponding dimeric ion at *m*/*z* 863.20 ([2 M–H]^−^), accompanied by comparable fragmentation behavior. The agreement in precursor ion masses and fragmentation behavior supports the interpretation of the present MS data. However, to strengthen structural assignment and to resolve potential positional isomerism, additional analytical strategies are warranted. In particular, high‐resolution MS/MS experiments employing stepped collision energies and detailed neutral loss analysis have been demonstrated to effectively differentiate flavone C‐glycoside isomers and sugar attachment patterns. Ultimately, unambiguous structural confirmation will require compound isolation followed by comprehensive NMR spectroscopic analysis (^1^H, ^13^C, HSQC, HMBC, and ROESY), which remains the gold standard for distinguishing C‐ versus O‐glycosylation and confirming aglycone substitution patterns.

Nevertheless, HPLC‐UV/VIS analysis revealed a significant difference in the distribution of the two compounds between EAV1 and EAV2 (Table 4). Specifically, the total peak area of the two flavonoid *C*‐glycosides in EAV1 was significantly greater than in EAV2 at the same measured concentration of 0.5 mg/mL (Figure 1), suggesting a higher content of these flavonoid *C*‐glycosides in EAV1. However, as noted above, comprehensive quantification requires isolation of the two compounds and construction of calibration curves.

**Table 4:** HPLC‐ESI‐MS/MS and HPLC‐UV/VIS analyses of major compounds in EAV samples.

Peak	Precursor (m/z)	Precursor type	Tentative classification	Ion fragmentation (MS/MS)	Total peak area in EAV1	Total peak area in EAV2
1st	863.20	[2 M‐H]^−^	Flavonoid glycoside	431 (100), 327 (27), 357 (6)	1542689	623936
2nd	431.10	[M‐H]^−^	Flavonoid glycoside	327 (100), 357 (12), 413 (1)		

### 3.3. Antibacterial Activity of AV Extracts Against *V. cholerae*

The antibacterial potential of EAV1 and EAV2 extracts against pathogenic *V. cholerae* strains was evaluated. Initially, we evaluated the antibacterial activity of the extracts using the disc diffusion assay. However, no zone of inhibition was observed to indicate the relative antibacterial activity of the extracts. This observation could be attributed to the solubility and rate of diffusion of the compounds in the agar medium [44]. Moreover, evaporation of the extract′s constituents can influence the results of the disc method. Therefore, we also evaluated the antimicrobial activity of EAV1 and EAV2 extracts using the broth microdilution assay by determining the MBC and MIC values (Table 5). We observed similar antibacterial activity between EAV1 and EAV2 extracts against the tested *V. cholerae* strains. Except *V. cholerae* P1418, both MIC and MBC values for EAV1 and EAV2 were 250 and 500 *μ*g/mL, respectively. For *V. cholerae* P1418, lower MIC (125 *μ*g/mL) and MBC (250 *μ*g/mL) values were observed for both EAV1 and EAV2. Although the observed MIC and MBC values showed moderate activity, the MBC/MIC ratio of 2 indicates bactericidal potential of the EAV extracts against *V. cholerae* [45].

**Table 5:** MBC and MIC values of EAV1 and EAV2 extracts against *V. cholerae*.

	EAV1		EAV2	
	MBC (*μ*g/mL)	MIC (*μ*g/mL)	MBC (*μ*g/mL)	MIC (*μ*g/mL)
P1418	250	125	250	125
14‐9/9	500	250	500	250
MO20	500	250	500	250
B0311	500	250	500	250

Flavonoid glycosides were identified as the major constituents in the EAV extracts. Flavonoid glycosides are compounds in which flavonoid aglycones are linked to different saccharide groups [46]. These compounds are widespread in plants, acting as antimicrobial compounds in response to plant pathogens [47]. Some flavonoid glycosides have been used as medicine and nutraceuticals due to their bioactivities, bioavailability, and low toxicities to humans [47]. The antimicrobial activity of flavonoid glycosides is attributed to the interaction of the compound with the bacterial membrane proteins or lipids resulting in membrane stabilization and disruption [48]. Flavonoid glycosides from *Graptophyllum glandulosum* have been reported to elicit antibacterial activity against multidrug‐resistant *V. cholerae* O1 [49]. These compounds damage the cytoplasmic membrane leading to efflux of nucleic acids. The same compounds extracted from *Maytenus buchananii* exhibited antibacterial activity against *V. cholerae* [50]. Blueberries, which are also rich in flavonol glycosides, were reported to elicit antimicrobial activity against pathogenic strains of *V. cholerae* [51]. Moreover, the quercetin‐glycoside rutin equally possesses antibacterial and antibiofilm properties against a multidrug‐resistant *Pseudomonas aeruginosa* [52] and drug‐resistant *Klebsiella pneumoniae* [53]. Flavonol glycosides from *Crotalaria maderensis* also elicit antibacterial and antioxidant properties against methicillin‐resistant *S. aureus* [54].

### 3.4. Inhibition of Biofilm Formation by EAV Extracts

Biofilms are communities of bacteria embedded in a self‐produced matrix that are either attached to a surface or as floating aggregates [55]. Biofilm formation by *V. cholerae* is linked to its pathogenesis. Biofilm‐associated cells are hyperinfectious, characterized by upregulated virulence factor production compared with planktonic cells [56].

EAV1 and EAV2 extracts were evaluated for their inhibitory effect on *V. cholerae* biofilms. In this experiment, we derived the MBIC_90%_, MBIC_50%_, and half‐maximal (50%) inhibitory concentration (IC_50_) [38]. MBIC_90%_ and MBIC_50%_ are defined as the concentration of the AV extracts resulting in at least 90% or 50% biofilm inhibition, respectively, whereas IC_50_ describes the estimated AV concentration to yield a 50% biofilm inhibition. MBIC values of EAV1 and EAV2 were compared (Table 6). In terms of MBIC_90%_, greater than 250 *μ*g/mL of the EAV extracts reduced at least 90% of biofilm growth. This was expected since these concentrations were regarded as the MIC and MBC of the EAV extracts.

**Table 6:** Minimum biofilm inhibitory concentration of EAV1 and EAV2 to *V. cholerae* biofilm.

	EAV1 (*μ*g/mL)			EAV2 (*μ*g/mL)		
	MBIC_90%_	MBIC_50%_	IC_50_	MBIC_90%_	MBIC_50%_	IC_50_
P1418	> 250	31.25	26.40	> 250	31.25	23.02
14‐9/9	> 250	125	69.89	> 250	125‐250	220.77
MO20	250	62.5–125	119.83	> 250	125–250	211.51
B0311	> 250	125–250	208.34	> 250	125–250	161.50

The IC_50_ was also derived to compare EAV1 and EAV2 biofilm inhibition activity against the tested *V. cholerae* strains. Overall, EAV1 demonstrated stronger biofilm inhibition than EAV2, with notably lower IC_50_ values across the tested *V. cholerae* strains, particularly against P1418. We also observed higher antibiofilm activities exhibited by EAV1 compared with EAV2 extract. This greater antibiofilm activity of EAV1 compared with EAV2 could be attributed to its higher TPC and TFC. In addition, the higher flavonoid C‐glycosides content in EAV1 than in EAV2 could have influenced the greater antibiofilm activity of EAV1.

Our present work focused on evaluating the bioactivity of the EAV crude extract to establish a baseline antibacterial and antibiofilm potential. While fractionation and purification may enhance the antibiofilm activity by concentrating the active constituents, synergistic interactions among multiple compounds in the crude extract may also contribute to the antibiofilm activity. For example, fractions (250 *μ*g/mL) from *Caesaria sylvestris* resulted in significant log reduction of viable cells of *Streptococcus mutans* biofilms while its crude leaf extract did not exhibit > 3 log reductions of viable bacteria indicating that the crude extracts (500 *μ*g/mL) did not present good biological activity [57]. Meanwhile, Assumpcao et al. [58] showed that crude extract of *Hypericum brasiliense* was more effective in eradicating the biofilms of *Staphylococcus* spp. compared with its fractions of uliginosin‐B and japonicin.

A limitation of this study is the absence of a positive control with known antibiofilm activity, for example rutin, quercetin, or resveratrol. Including a positive control in future studies would allow more direct benchmarking of the EAV extract antibiofilm activity against established antibiofilm compounds. However, our results showed strong antibiofilm activity of EAV extracts against *V. cholerae* biofilm formation. Reported MBIC_90%_ of pure clove (*Syzygium aromaticum*) and eugenol essential oils against *V. cholerae* biofilms were at 3125 *μ*g/mL and 625 *μ*g/mL, respectively [59]. Moreover, MBIC_50%_ values for *H. antidysenterica*, *C. sinensis*, *E. scaber*, and *C. asiatica* extracts were at 600, 500, 1000, and 2000 *μ*g/mL, respectively [10]. These reported MBIC_90%_ and MBIC_50%_ were higher compared with the MBIC values observed with EAV extracts. Our findings suggest that extracts from *Andropogon* spp. are potential sources of strong biofilm‐inhibiting compounds against *V. cholerae* and potentially to other biofilm‐forming pathogens.

### 3.5. Eradication of Preformed Biofilms by EAV Extracts

The biofilm eradication activity of EAV1 and EAV2 against *V. cholerae* preformed biofilm was evaluated (Table 7). MBEC_50%_ refers to the minimum EAV concentration to elicit at least 50% eradication of the 24‐h preformed biofilms. EAV1 showed stronger biofilm eradication activity than EAV2, with both extracts showing MBEC_50%_ values from 250–300 *μ*g/mL against P1418 and 14‐9/9, whereas MO20 and B0311 required concentrations exceeding 500 *μ*g/mL.

**Table 7:** Minimum biofilm eradication concentration of EAV1 and EAV2 to *V. cholerae*‐preformed biofilm.

	EAV1		EAV2	
	MBEC_50%_ (*μ*g/mL)	IC_50_ (*μ*g/mL)	MBEC_50%_ (*μ*g/mL)	IC_50_ (*μ*g/mL)
P1418	250	130.04	250	153.84
14‐9/9	300	257.36	300	264.59
MO20	> 500	> 500	> 500	> 500
B0311	> 500	> 500	> 500	> 500

To allow comparison of the biofilm eradication efficacy between EAV1 and EAV2, the IC_50_ was also evaluated. IC_50_ values indicated greater susceptibility of P1418 and 14‐9.9 compared with the O139 strains. Higher concentrations of EAV extracts were required to eradicate at least 50% of preformed biofilms compared with at least 50% biofilm inhibition, indicating that biofilm formation by *V. cholerae* provides resistance to antimicrobials, such as in this study, resistance to the EAV extracts. Consistent with the MBIC assay, EAV1 showed stronger activity overall.

As a virulence factor, biofilm formation provides protection to *V. cholerae* cells by enhancing resistance to antimicrobials and host immune responses [7]. For example, *V. cholerae* biofilm‐associated cells exhibit greater resistance to five different classes of antibiotics compared with the planktonic cells [60]. Similarly, resistance to silver nanoparticle has been reported in *V. cholerae* biofilms [61]. The higher MBEC_50%_ and IC_50_ values of EAV1 and EAV2 obtained from the MBEC assay compared with the MBIC assay confirmed that *V. cholerae* biofilms are indeed a physical barrier against antimicrobial agents. Previous studies have also observed similar reduced antibiofilm efficacy of ethanolic plant extracts against the established biofilms of *V. cholerae* [62, 63].

### 3.6. Gene Expression Analysis

**Figure 2:**
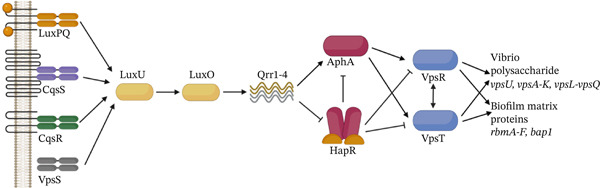
Quorum sensing regulation of biofilm formation in *V. cholerae*. Created by http://Biorender.com.

*V. cholerae* biofilm formation is primarily regulated by its QS system [5] (Figure 2). In contrast, *V. cholerae* initiates biofilm formation under LCD conditions. At LCD, the QS master regulator AphA activates the biofilm regulators VpsT and VpsR, which in turn promote the production of VPS and biofilm matrix proteins [6]. These components are essential for the initiation and maturation of *V. cholerae* biofilms. Under high cell density (HCD), these positive biofilm regulators are repressed by the HCD master QS regulator HapR, thereby downregulating biofilm formation. Strains harboring a nonfunctional HapR are locked in the LCD state, resulting in markedly higher biofilm formation [34]. Although we included both HapR‐positive and HapR‐negative strains to capture potential differences in biofilm regulation, no clear association was observed between HapR variation and the antibiofilm activities of EAV1 and EAV2 extracts. This suggests that the antibiofilm activity of the extracts may act through mechanisms independent of HapR‐mediated QS regulation.

QS disruption or QQ is an effective way to control bacterial virulence [64]. This process refers to the interference of the QS system, which inhibits bacterial communication using chemical or enzymatic means and therefore reduces the behaviors associated with QS [65]. This strategy does not intentionally kill the bacteria but instead inhibits the production of virulence factors, making the pathogen less virulent [66]. In screening for potential QQ compounds, MIC assays are performed to ensure that the compound has no effect on the bacterial growth [64]. The reduced biofilms below MIC levels of both EAV1 and EAV2 indicate a potential QQ activity of *Andropogon* spp. extracts in terms of biofilm inhibition in *V. cholerae*.

**Figure 3:**
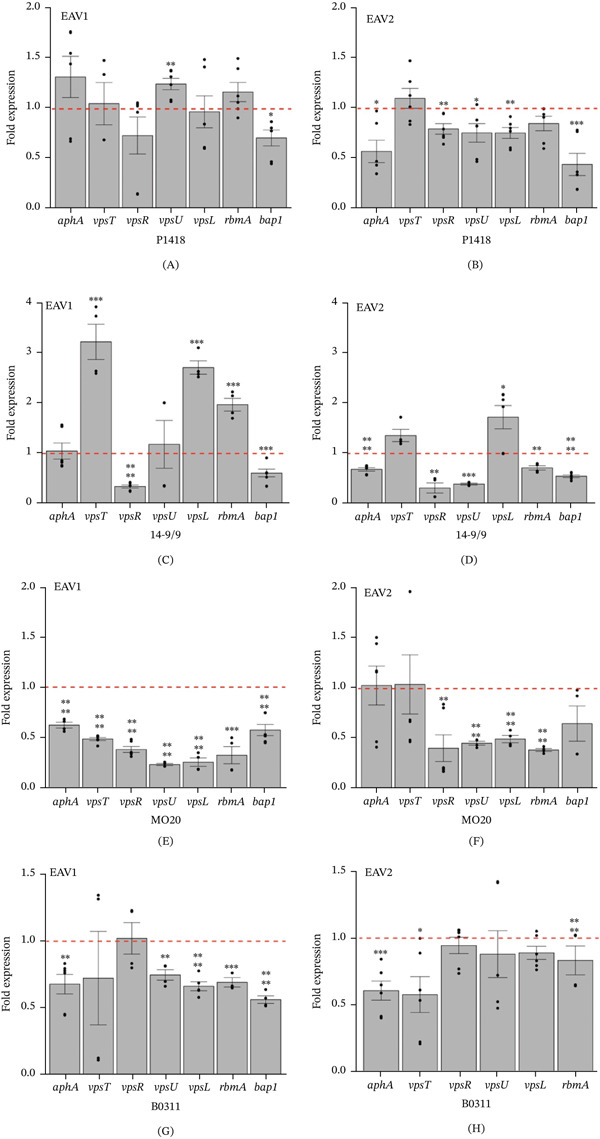
Quantitative real‐time PCR analysis of *V. cholerae* strains grown under 0.75× MIC of EAV1 and EAV2 extract. (A,C,E,G) Gene expression in EAV1 treatment and (B,D,F,H) gene expression in EAV2 treatment. *V. cholerae* strains are indicated below each panel. Red broken lines indicate expression of control treatment (without extract) equal to 1. Error bars indicate standard error. Statistical significance was calculated using Student′s *t*‐test. Asterisk: ∗, *p* < 0.05; ∗∗, *p* < 0.01; ∗∗∗, *p* < 0.005; ∗∗∗∗, *p* < 0.001.

*V. cholerae* biofilms are composed of VPS and matrix proteins encoded by the *vps-rbm* operon. Figure 3 shows the fold‐change expression of biofilm regulators and matrix‐associated genes (*aphA*, *vpsT*, *vpsR*, *vpsU*, *vpsL*, *rbmA*, and *bap1*) in four *V. cholerae* strains exposed to EAV1 and EAV2. In strain P1418, EAV1 significantly upregulated *vpsU* (1.248‐fold) while downregulating *bap1* (0.709‐fold) (Figure 3A). More pronounced repression was observed under EAV2, where *aphA* (0.571‐fold)*, vpsR* (0.796‐fold), *vpsU* (0.755‐fold), *vpsL* (0.755‐fold), and *bap1* (0.44‐fold) were significantly reduced (Figure 3B). In strain 14‐9/9, EAV1 resulted in the significant upregulation of *vpsT* (3.229‐fold), *vpsL* (2.715‐fold), and *rbmA* (1.973‐fold) and downregulation of *vpsR* (0.341‐fold) and *bap1* (0.610‐fold) (Figure 3C). Upregulation of *vpsL* (1.724‐fold) and significant downregulation of *aphA* (0.683‐fold), *vpsR* (0.312‐fold), *vpsU* (0.390‐fold), *rbmA* (0.713‐fold), and *bap1* (0.547‐fold) were observed in EAV2 (Figure 3D). For MO20 strain, EAV1 significantly downregulated the evaluated genes (0.227 to 0.621‐fold) (Figure 3E) while EAV2 also downregulated *vpsR* (0.404‐fold), *vpsU* (0.455‐fold), *vpsL* (0.496‐fold), *rbmA* (0.387‐fold) (Figure 3F). Similarly, in B0311, EAV1 significantly downregulated the expression levels of *aphA* (0.679‐fold), *vpsU* (0.748‐fold), *vpsL* (0.634‐fold), *rbmA* (0.714‐fold), and *bap1* (0.563‐fold) (Figure 3G). Significant downregulation of *aphA* (0.607‐fold), *vpsT* (0.577‐fold), and *bap1* (0.884‐fold) expression by EAV2 was also observed (Figure 3H). Expression levels of the positive biofilm regulators *aphA*, *vpsT*, and *vpsR* varied among strains but generally showed reduced expression under EAV treatment. As observed, downregulation of these transcriptional regulators resulted in the downregulation of biofilm‐associated genes, thereby reducing biofilm formation. Both EAV1 and EAV2 may influence biofilm formation via mechanisms that could include modulation of QS‐related regulators. Recent research has shown that phenolic compounds derived from plants inhibit QS‐regulated phenotypes [67]. These phenolic compounds and those compounds in the EAV extracts target the expression of QS‐related genes [68–70]. In addition, flavonoids, a class of phenolic compounds, act by an allosteric mechanism by binding to a site of the QS‐receptor without accessing the autoinducer binding pocket in the autoinducer‐binding receptors of the QS system [71]. This noncompetitive QS‐inhibition by flavonoids is attributed to the two hydroxyl moieties in the flavone A‐ring backbone. Flavonoid glycosides also exhibit anti‐QS activity as exemplified by the inhibition of violacein production in *Chromobacterium violaceum* and pyocyanin production inhibition in *P. aeruginosa* when treated with flavonoid glycosides from *Psidium quajava* extracts [72]. Similarly, the quercetin‐glycoside rutin showed antibiofilm activities against the multidrug resistant *P. aeruginosa* biofilms [52]. Glycosylflavonoids from *Cecropia pachystachya* also showed QS‐inhibition potential as these compounds inhibit violacein production in *C. violaceum* and bioluminescence in *E. coli* [73].

The production of *V. cholerae* biofilm components is regulated by the *vps* operon [74]. The VPS is encoded by the *vps-I* (*vpsU*, *vpsA-vpsK*) and *vps-II* (*vpsL-vpsQ*) gene clusters initiated by *vpsU* and *vpsL*, respectively. The biofilm matrix proteins are encoded in the *rbm* (*rbmA-rbmF*) gene cluster initiated by *rbmA* [74–76]. Another biofilm matrix protein, known as the biofilm‐associated protein Bap1, is encoded by the *bap1* gene. The *vps* genes are required for the maturation of the biofilms while the matrix proteins provide and maintain the structural integrity of biofilms [77]. The initiation and maturation of *V. cholerae* biofilm formation is characterized by sequential production of biofilm components [78, 79]. After initial attachment, VPS is excreted from the cell surface and VPS production occurs throughout the biofilm formation. Then, RbmA (biofilm structure modulator A) accumulates on the cell surface. RbmA also facilitates the cell‐to‐cell adhesion between founder and daughter cells. Bap1 is then excreted between cells and on the substrate and gradually accumulates radially on nearby surfaces. As the biofilm develops, VPS, RbmC, and Bap1 form the matrix that grows as cells divide. For genes involved in *Vibrio* polysaccharide production, *vpsU* expression was significantly reduced by EAV treatment compared with *vpsL* (Figure 3). Although *bap1*, which encodes a biofilm‐associated protein, is located outside the *vps-rbm* operon, its expression was consistently reduced across all strains treated with either EAV1 or EAV2. Notably, the O139 strains MO20 and B0311 exhibited greater reductions in biofilm‐associated gene expression in response to EAV extracts compared with O1 El Tor strains.

In the present study, the expression of seven biofilm‐associated genes in *V. cholerae* were studied under EAV treatment. The reduction in the expression of *aphA, vpsT,* and *vpsR* has led to the reduced *vpsU*, *vpsL*, *rbmA*, and *bap1* expression in some *V. cholerae* strains. This resulted in the reduced biofilm formation by *V. cholerae* upon exposure to the sub‐MIC levels of EAV1 and EAV2 extracts. Although EAV treatment showed no significant difference or increase in the expression of some biofilm‐associated genes, the expression of *bap1* was consistently downregulated in all strains. Bap1 production is important in the initiation and attachment of *V. cholerae* during biofilm formation [78, 79]. In addition, Bap1 is required for the maintenance of the mechanical strength and hydrophobicity of pellicles, a biofilm formed in the air‐liquid interface [80]. In *V. cholerae* Δ*bap1* deletion mutant, altered pellicle structures, colony morphologies, and biofilm structures were observed [76]. Furthermore, this defect in the biofilm formation of the Δ*bap1* deletion mutant is independent of reduced *vps* transcription. Overall, our findings demonstrate that EAV extracts inhibit *V. cholerae* biofilm formation by modulating the expression of key biofilm regulators and biofilm‐associated genes.

### 3.7. Swimming Motility

**Figure 4:**
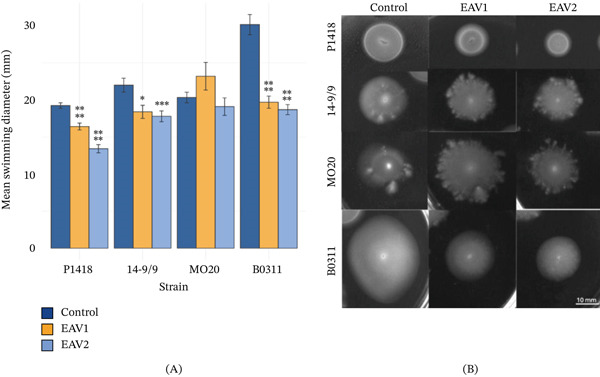
Swimming motility of *V. cholerae* strains in 0.75× MIC of EAV1 and EAV2 extract. (A) Swimming motility diameters of *V. cholerae* strains in 0.75× MIC of EAV1 and EAV2 extract. Error bars indicate standard error. Statistical significance was calculated using Student′s *t*‐test. Asterisk: ∗, *p* < 0.05; ∗∗, *p* < 0.01; ∗∗∗, *p* < 0.005; ∗∗∗∗, *p* < 0.001. (B) Representative swimming motility images of *V. cholerae* strains treated with 0.75× MIC of EAV1 and EAV2 extracts.

Motility is important in the survival of *V. cholerae* in the aquatic environments and pathogenicity. In *V. cholerae*, motility facilitates the initial attachment of cells for biofilm formation. Thus, both motility and biofilm formation are considered virulence factors of V*. cholerae*, in addition to cholera toxin and pilus production. The impact of EAV extracts on the swimming motility of *V. cholerae* was also evaluated. EAV1 and EAV2 extracts suppress the swimming motility in most *V. cholerae* strains, whereas MO20 appears unaffected (Figure 4). Although the swarming motility assay using M9 minimal medium with 0.5% agar was also performed (data not shown), swarming was not observed even after extended incubation after 48 h. Nevertheless, the reduction in swimming diameter suggests an inhibitory effect of the extracts on *V. cholerae* motility, which could be linked to impaired flagellar function or QS. The antimotility activity of the EAV extracts could be attributed to the phytochemicals present. For example, some glycosylated flavanones exhibit significant inhibitory effects on the swimming motility in *Yersinia enterocolitica* [81]. Flavonols, such as quercetin‐0‐ß‐glucopyranoside, also inhibit swarming motilities in *P. aeruginosa* and *Serratia marcescens* [82] through binding to the QS regulator, LasR. Similar antimotility signatures of ethanolic spice extracts were also reported against pathogenic and nonpathogenic *V. cholerae* strains [62]. The reduction in swimming diameter suggests an inhibitory effect of the EAV extracts on *V. cholerae* swimming motility, which could be linked to impaired flagellar function or QS.

### 3.8. Microscopic Analysis

**Figure 5:**
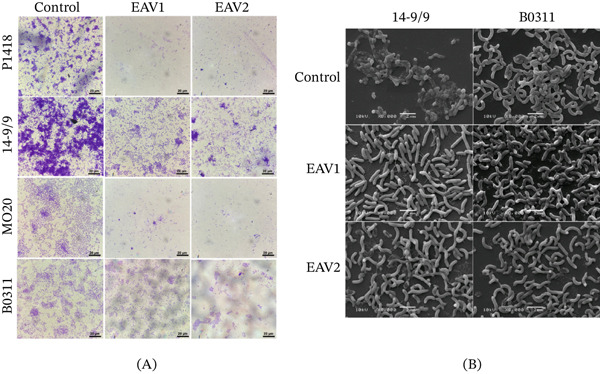
Microscopic analysis results *V. cholerae* biofilms treated with 0.75× MIC of EAV1 and EAV2 extract. (A) Compound light microscopy at 1000× magnification. (B) Representative SEM images of *V. cholerae* 14‐9/9 and B0311 biofilms.

A light microscopy was performed to confirm the biofilm inhibition activities of the EAV extracts against *V. cholerae* biofilms (Figure 5A). In the untreated control, dense multilayered biofilm structures were observed. In both P1418 and 14‐9/9, biofilms are characterized by thick cell clusters and microcolony formation, whereas MO20 and B0311 exhibited confluent cell clusters. In contrast, *V. cholerae* biofilms treated with EAV1 and EAV2 extracts at 0.75× MIC exhibited a marked reduction in biofilm intensity compared with the control. The biofilm appeared thinner, less confluent, and scattered colonies.

SEM analyses were performed for the HapR‐positive strain 14‐9/9 and HapR‐negative strain B0311 to determine morphological alterations in *V. cholerae* biofilms following treatment of EAV extracts (Figure 5B). In 14‐9/9 untreated control, cells are covered with thick layers of extracellular matrix. In contrast, treatment with 0.75× MIC EAV1 and EAV2 extracts significantly eradicated the extracellular matrix as indicated by the exposed cells and remnants of the biofilm matrix on the glass surface. Moreover, some cells appear moderately shriveled indicating modifications in the cellular membrane. For B0311 control, the distinct thick biofilms were not observed. Cells were full, with clear and smooth surfaces. Similar to treated 14‐9/9 biofilms, treated B0311 cells exhibited moderately shriveled appearance.

## 4. Conclusion

In conclusion, bluestem (*Andropogon* spp.) extracts exhibited antibacterial and antibiofilm activity against pathogenic *V. cholerae* strains. The phenolic and flavonoid compounds, specifically flavonoid C‐glycosides, present in the extract elicit the antibacterial, biofilm inhibition, and antimotility activities. These results show the potential biofilm inhibition mechanism by EAV1 and EAV2 extracts in *V. cholerae*, through inhibition of biofilm‐associated gene expression, which could facilitate the development of new strategies for cholera infection control. In addition, this study demonstrated the potential of plant species with invasive tendencies to be a source of antibacterial and antibiofilm compounds against different clinically important pathogens such as *V. cholerae*.

## Author Contributions

J.C.C. and N.V.Q. conceptualized and investigated the study. J.C.C. and N.V.Q. worked in data curation. J.C.C. prepared the original draft. J.C.C., T.S., T.D.X., and N.V.Q. reviewed and edited the manuscript.

## Funding

No funding was received for this manuscript.

## Disclosure

All authors have read and agreed to the published version of the manuscript.

## Conflicts of Interest

The authors declare no conflicts of interest.

## Supporting information


**Supporting Information** Additional supporting information can be found online in the Supporting Information section. Figure S1 and Figure S2 present the MS/MS spectra of the two major compounds detected in the HPLC‐UV chromatogram. These peaks correspond to the two major chromatographic signals observed in Figure 1. Figure S1 shows the MS/MS spectrum of the compound corresponding to the first elution peak, detected at a retention time of 9.05 min. Figure S2 illustrates the MS/MS spectrum of the compound corresponding to the second elution peak, detected at a retention time of 9.17 min.

## Data Availability

The data presented in this study are available upon request from the corresponding author.
