# Supplementary material for: Antibacterial and Antibiofilm Activities of Bluestem (Andropogon spp.) Extracts against Vibrio cholerae
Source: Int J Microbiol. 2026 May 3;2026:9982329. doi: 10.1155/ijm/9982329 (PMC13136520; doi:10.1155/ijm/9982329)
Supplement: Supplementary file 1 — Supporting Information Additional supporting information can be found online in the Supporting Information section. Figure S1 and Figure S2 present the MS/MS spectra of the two major compounds detected in the HPLC‐UV chromatogram. These peaks correspond to the two major chromatographic signals observed in Figure 1. Figure S1 shows the MS/MS spectrum of the compound corresponding to the first elution peak, detected at a retention time of 9.05 min. Figure S2 illustrates the MS/MS spectrum of the compound corresponding to the second elution peak, detected at a retention time of 9.17 min. [file IJM-2026-9982329-s001.docx]

Absorbance (%)

**Fig. S1. MS/MS spectrum of the compound corresponding to the 1st HPLC-UV elution peak**, collected at a retention time of 9.05 min ([2M-H]^-^: 863.20 m/z). This peak represents the earlier of the two major chromatographic peaks observed in Fig. 1 (RT 7.35 min).

Absorbance (%)

**Fig. S2. MS/MS spectrum of the compound corresponding to the 2nd HPLC-UV elution peak**, collected at a retention time of 9.17 min (([M-H]^-^: 431.10 m/z). This peak represents the earlier of the two major chromatographic peaks observed in Fig. 1 (RT 7.57 min).
